# Advantages of Phosphodiesterase Type 5 Inhibitors in the Management of Glucose Metabolism Disorders: A Clinical and Translational Issue

**DOI:** 10.1155/2020/7078108

**Published:** 2020-07-28

**Authors:** Cristina Antinozzi, Paolo Sgrò, Luigi Di Luigi

**Affiliations:** Unit of Endocrinology, Department of Movement, Human and Health Sciences, University of Rome “Foro Italico”, 00135 Rome, Italy

## Abstract

Among metabolic diseases, carbohydrate metabolism disorders are the most widespread. The most common glucose pathological conditions are acquired and may increase the risk of type 2 diabetes, obesity, heart diseases, stroke, and kidney insufficiency. Phosphodiesterase type 5 inhibitors (PDE5i) have long been used as an effective therapeutic option for the treatment of erectile dysfunction (ED). Different studies have demonstrated that PDE5i, by sensitizing insulin target tissues to insulin, play an important role in controlling the action of insulin and glucose metabolism, highlighting the protective action of these drugs against metabolic diseases. In this review, we report the latest knowledge about the role of PDE5i in the metabolic diseases of insulin resistance and type 2 diabetes, highlighting clinical aspects and potential treatment approaches. Although various encouraging data are available, further in vivo and in vitro studies are required to elucidate the mechanism of action and their clinical application in humans.

## 1. Introduction

Phosphodiesterases (PDEs) are a family of 11 enzyme isoforms, which hydrolyze cyclic nucleotides to regulate intracellular levels of the second messengers, cyclic adenosine monophosphate (cAMP) and cyclic guanosine monophosphate (cGMP), modulating the duration and intensity of different intracellular functions [1, 2]. Among these, the isoform 5 (PDE5) hydrolyzes cGMP in an inactive 5′-GMP form, with consequent different cellular responses [3]. The inhibition of this enzyme increases the intracellular level of cGMP, prolonging specific cGMP-related cellular responses. Different drugs inhibiting PDE5 (PDE5i) exist, of which the main substances commercially available are sildenafil, vardenafil, tadalafil, and the more recently approved avanafil [1]. The PDE5i activity has long been used as an effective therapeutic option for the treatment of erectile dysfunction (ED) and for other diseases (i.e., pulmonary hypertension and Raynaud's phenomenon) [3, 4]. However, recent studies have suggested that PDE5i may also be useful in other pathological conditions such as cardiovascular diseases (i.e., coronary artery disease and hypertension) [5–12], autoimmune diseases, and endocrine-metabolic disorders [13–16].

In particular, endocrine-metabolic disorders define a cluster of pathological conditions, which may also greatly increase health risk for cardiovascular diseases [17]. Insulin resistance and obesity are considered the core of most cases of metabolic syndrome and cardiovascular complications where many factors (e.g., endothelial dysfunction, polycystic ovary syndrome (PCOS), hypogonadism, genetic susceptibility, hypertension, and chronic stress) may favor such complications [18]. These metabolic diseases often also induce an alteration of inflammatory status, resulting in increased reactive oxygen species (ROS) production [19].

In this context, due to their observed antioxidant and anti-inflammatory properties, several studies have highlighted the protective action of PDE5i against endothelial dysfunction and cardiac ischemic/reperfusion [2]. Furthermore, in vivo and in vitro studies have shown how PDE5 inhibition may ameliorate insulin resistance in diabetic mice [20–22] and sensitize skeletal muscle cells, adipocytes, and endothelial cells to insulin action, thus improving insulin resistance [13, 23–26].

Based on recent in vivo and in vitro findings, this review summarizes the molecular mechanisms that underline the action of PDE5 on intracellular metabolic pathways, the effects of the PDE5i, and the association between PDE5i and metabolic disorders, focusing on glucose metabolism disorders and on the potential therapeutic role of PDE5i to treat insulin resistance and type 2 diabetes.

## 2. PDE5-Related Biochemical Pathways

The biochemical pathways involving PDE5 have largely been studied in smooth muscle cells, investigating the biological basis and mechanism of PDE5i in ED treatment [27]. This mechanism requires the release of nitric oxide (NO) by endothelial cells, its diffusion to smooth muscle cells, and the activation by NO of guanylate cyclase enzyme. The interaction of NO with guanylyl cyclase induces a conformational change in this enzyme, which results in the catalytic production of 3′-5′-cyclic guanosine monophosphate from guanosine 5′-triphosphate [28]. Cyclic GMP activates cGMP-dependent protein kinase (PKG), which induces the activation of different substrates. These interactions induce the reduction of intracellular calcium levels and, consequently, relaxation of arterial and trabecular smooth muscle, leading to arterial dilatation, venous constriction, and penile rigidity [29]. PDE5, which is abundant in vascular smooth muscle cells (VSMC), normally inhibits this process by degrading cGMP. The balance between cGMP synthesis by guanylyl cyclase and cGMP hydrolysis by PDE5 regulates cGMP levels in the corpora cavernosa [30]. Various pathological conditions, such as structural and functional endothelium deterioration, insufficient NO production, excessive cGMP hydrolysis by PDE5, and reduction of the conformity of the VSMC and of the connective tissue of the penis (or a combination of the above), can compromise this process.

PDE5i has the capacity to lower the activity of PDE5 by competing with cGMP, subsequently raising its level in the cells [1, 2, 28–30]; thus, the action of the PDE5i is useful to promote the accumulation of cGMP and to potentiate the effect of NO [1].

## 3. Molecular Basis of PDE5 Inhibitor Action on Insulin Target Tissues

Besides the abovementioned effects, data suggest that PDE5i could target insulin-sensitive cells, such as skeletal muscle cells, adipocytes, hepatocytes, and endothelial cells, through an insulin-like effect [13, 22–24, 31, 32]. Moreover, PDE5 and nitric oxide synthase (NOS) have been shown to be highly expressed in different tissues commonly involved in glucose metabolism regulation [33–35].

**Figure 1:**
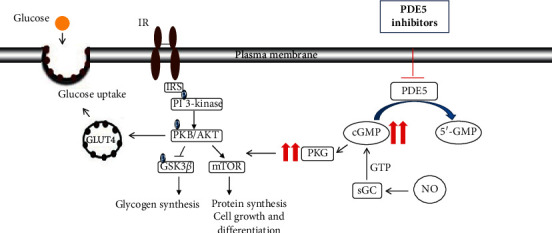
Schematic representation of PDE5i action in insulin-related pathways. PKG activated by cGMP exerts positive effects on IRS downstream effectors. The phosphorylation of PKB/AKT induces the translocation of GLUT4 vesicles on plasma membrane, favoring glucose uptake and activation of downstream signaling involved in protein synthesis, cell growth, and differentiation. The balance between cGMP synthesis (induced by the interaction of NO with sGC) and cGMP hydrolysis (by PDE5) regulates cGMP levels. The action of the PDE5i promotes the accumulation of cGMP. IR: insulin receptor; NO: nitric oxide; sGC: guanylyl cyclase; PDE5: phosphodiesterase type 5; GMP: guanosine monophosphate; cGMP: cyclic guanosine monophosphate; IRS: insulin receptor substrate; PI3K: phosphatidilinositol 3-kinase; PKB/AKT: protein kinase B/AKT; and mTOR: mammalian target of ramapycin.

Molecular studies carried out in the skeletal muscle have demonstrated that the inhibition of PDE5 may activate the same downstream pathways related to the action of insulin [13, 24, 31] (Figure 1). In particular, studies in human skeletal muscle cells maintained in normoglycemic conditions showed that a single dose of tadalafil induced, over a few minutes, the following: (a) an increase of glucose transporter (GLUT) 4 gene expression; (b) activation of the peroxisome proliferator-activated receptor gamma (PPAR*γ*) and insulin receptor substrate (IRS)-1; (c) stimulation of the phosphorylation of IRS-1, protein-kinase B (PKB/AKT), mammalian target of rapamycin (mTOR), and glycogen synthase kinase 3 beta (GSK3*β*); and (d) translocation on the plasmatic membrane of GLUT4, caveolin-1, and flotillin-1 [13]. Other studies have demonstrated the involvement of NO/cGMP-related pathways in metabolism regulation, both in skeletal muscle cells and in other insulin-dependent cells; particularly adipocytes and endothelium [36]. NO and cGMP activate PKG, playing an important role in mediating insulin-induced effects, especially favoring glucose uptake, insulin-related pathway activation (e.g., AKT and IRS-1), and GLUT4 translocation [22, 25, 37, 38], as well as inducing the activation of sirtuin-1 (SIRT1), a key sensor of cellular energy status [39–42]. Furthermore, it has been reported that NO release induces 5-AMP activated protein kinase (AMPK) [38], an enzyme which promotes glucose transport and fatty acid oxidation in insulin-responsive tissues [43, 44] and increases GLUT4, mitochondrial proteins, and several metabolic enzymes expression after metabolic challenges [45–48]. Thus, by interfering at various levels with insulin-related pathways, PDE5i may have different pharmacological effects influencing the regulation of glucose metabolism.

## 4. PDE5 Inhibitors and Insulin Resistance

**Figure 2:**
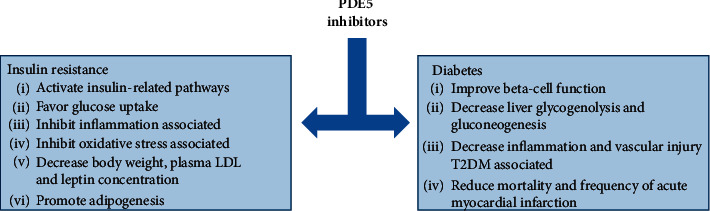
Pivotal roles of PDE5i in glucose metabolic disorders. Schematic image summarizing the main effects of PDE5 inhibitors in insulin resistance and diabetes. LDL: low-density lipoprotein and T2DM: type 2 diabetes mellitus.

Insulin resistance (IR) is a pathological condition in which the action of insulin, which aims to facilitate glucose uptake and metabolism in peripheral tissues, is reduced [49]. IR is a basic feature of the etiology in different metabolic disorders and has been linked to a wide range of other pathophysiologic sequelae, including type 2 diabetes, hypertension, hyperlipidemia, and obesity [49]. Evidence suggests that, by targeting insulin-sensitive cells, PDE5 inhibition may contribute to the improvement of insulin resistance (Figure 2); in particular, in skeletal muscles, adipocytes, hepatocytes, and endothelial cells [13, 22, 24, 30, 32]. As previously mentioned, different studies have demonstrated the direct effect of PDE5 inhibition on insulin pathway activation in skeletal muscle cells [13], suggesting a pivotal role of PDE5 in glucose metabolism regulation. To evaluate the functional role of PDE5 in the muscle tissue, Liu at al. performed molecular studies on differentiated mice muscle cells maintained in hyperglycemic conditions and explained why counteracting PDE5 may be a good strategy for ameliorating insulin sensitivity [49]. The authors demonstrated that, among all the characterized isoforms [50], the overexpression of PDE5A negatively regulated insulin signaling, with a decrease of the activation of insulin pathways and an inhibition of glucose uptake. Moreover, the authors suggested that this mechanism involves endoplasmic reticulum (ER) and the proteasome activity, two primary elements which regulate the synthesis, viability, folding, and assembly of all secretory, cellular, and membrane proteins [49].

Many studies have reported that NO signaling and its downstream effectors could play an important role in the improvement of inflammation and peripheral insulin resistance [51]. Furthermore, insulin also stimulates NO production in multiple organs involved in glucose metabolism [26, 51].

In endothelial cells maintained in growth conditions mimicking insulin resistance, it has been demonstrated that sildenafil improved the NOS activity by partially involving the activation of the phosphatidylinositol 3-kinase (PI3K) pathway [26]. Furthermore, in hyperglycemic conditions, when e-NOS and AKT-1 activation were reduced, sildenafil treatment restored normal protein function. Interestingly, the oxidative stress induced by hyperglycaemia in endothelial cells was also reduced by sildenafil treatment [26].

Different data have highlighted the role of the NO/cGMP/PKG pathway in adipocytes as well, given that PDE5 is highly expressed in these cells [22, 23]. Studies carried out in 3T3L1 mice preadipocyte cells demonstrated that chronic treatment with sildenafil for 8 days significantly promoted adipogenesis, increasing lipid droplet and triglyceride content. Moreover, sildenafil treatment increased the expression of the genes and proteins aP2, GLUT4, CCAAT enhancer-binding proteins (C/EBP) *α* and *β*, and PPAR*γ* and upregulated glucose uptake through PKG activation [52]. Finally, PDE5 inhibitors can promote adipogenesis, interfere with adipokine secretion, decrease inflammatory marker expression, and increase the thermogenic potential of the white adipose tissue and brown adipocyte differentiation [53].

These experiments have been confirmed in animal models, where it has been demonstrated that both acute and chronic treatments with sildenafil could improve insulin resistance and the associated endothelial dysfunction [54–56]. In particular, Ayala et al. demonstrated that mice fed an high-fat diet (HFD) treated with sildenafil (12 mg/kg/d for 12 weeks) showed a decreased weight gain and a 30% decrease in fat mass, compared with vehicle treatment [57]; furthermore, these mice showed an IR reduction, despite elevated daily food intake.

Different studies performed in diet-induced obese animal models have investigated the effects of a new PDE5i, DA-8159, as well as the PDE5i udenafil. In these studies, the authors demonstrated that the oral administration of PDE5i ameliorated body weight, plasma cholesterol and triglyceride levels, visceral fat mass, cumulative caloric intake, and plasma leptin concentration in comparison with vehicle-treated mice [58, 59].

In addition, a high-fat diet is able to induce IR in the liver and the proinflammatory activation of Kupffer cells, the resident macrophages of the liver [37]. Furthermore, an event often observed in association with obesity is a reduction in NO vascular content, which may lead to predisposition to increased endothelial and Kupffer cell inflammation, thrombosis, and vasoconstriction [60, 61]. Thus, targeting NO may represent a good strategy for ameliorating the inflammation induced by nutrient excess. In this context, in NO-/- mice fed with a low-fat diet, the authors showed that liver inflammation and IR induced by deficiency of NO/cGMP were comparable with that induced by a high-fat diet. Furthermore, they observed that these effects were prevented by daily oral dosing of sildenafil, supporting the hypothesis of the pivotal role of NO/cGMP signaling in protecting against liver IR and inflammation associated with diet-induced obesity [37, 62]. The potential usefulness of PDE5i administration in IR conditions and obesity IR-associated has also been partially demonstrated in humans, although further studies are warranted. In a randomized, double-blind trial in subjects with severe obesity (BMI ≥ 36.2 kg/m^2^) and elevated fasting insulin levels, treatment with tadalafil (20 mg/d) improved IR and beta-cell function, compared to placebo [63].

## 5. PDE5 Inhibitors and Diabetes

Type 2 diabetes mellitus (T2DM) represents the predominant type of diabetes, which accounts for nearly 90% of the population with diabetes. It is characterized by high blood sugar and insulin resistance [64]. In this population, PDE5i (particularly tadalafil and sildenafil) have been proven not only to be safe in diabetic men experiencing ED [59, 65, 66] but also to directly control metabolism regulation as T2DM tends to ameliorate after specific PDE5 inhibition (Figure 2) [20, 22].

Several studies have focused on the use of the PDE5i to treat inflammation, vascular injury, and hepatic diseases often associated with diabetes in both animal models and humans [67–69].

In fact, the hyperglycemic condition in T2DM patients is often associated with systemic inflammation [70, 71]. This is mainly due to an increase of inflammatory cells binding the endothelium [72] and to an increase of cytokine and chemokine production by peripheral blood mononuclear cells (PBMCs). In this context, Varma et al. demonstrated that treatment with tadalafil for 28 days in T2DM db/db mice significantly reduced the levels of the proinflammatory cytokines tumor necrosis factor (TNF)-*α* and interleukin (IL)-1*β* and increased that of anti-inflammatory IL-10 [16]. Analogous results were obtained in diet-induced obese (DIO) mice [69] where the authors showed that the combined therapy of PDE5i with leucine administration amplified the effects of PDE5 inhibition, resulting in fatty acid oxidation activation, insulin sensitivity improvement, and reversal of hepatic steatosis. Furthermore, at the same time, the activation of PKG significantly reduced the levels of proinflammatory cytokines commonly upregulated in diabetes [69].

Preclinical studies in patients affected by T2DM with cardiovascular complications showed an improvement in diabetic cardiomyopathy following chronic treatment with sildenafil 100 mg/d [14], as well as serum decreases of C-X-motif chemokine 10 (CXCL10) [6]—an important trigger of inflammation—and the cytokine IL-8, which is responsible for a wide range of inflammatory processes and vascular bed injury [5]. Other studies performed in T2DM patients have reported the advantage of sildenafil treatment for diabetic neuropathy and vasculopathy prevention [73] and demonstrated a significant improvement of endothelial function following both acute and chronic sildenafil administration (100 mg/d for 3 days and 25 mg/d for 4 weeks, respectively) [73–75]. Grover-Paez et al. demonstrated that daily treatment with sildenafil (50 mg/d for 30 days) improved both endothelial functional and reduced albuminuria and glycated hemoglobin (HbA1c), suggesting an effect of PDE5i also on endothelial cells [76]. Moreover, an in vivo study conducted on 5956 T2DM patients demonstrated a significant reduction of mortality and a lower frequency of acute myocardial infarction in subjects taking PDE5i [77]. In postmenopausal females with T2DM, an improvement in metabolic parameters after tadalafil administration was reported; in particular, patients exhibited a lower permeability surface area for glucose (PSglu) and an increase of lactate concentration both in muscle and adipose tissues [78].

A recent randomized study performed on T2DM patients and db/db mice receiving sildenafil (100 mg/d) or placebo for 12 weeks showed significant upregulation of miR-22-3p and SIRT1 protein expression [79]. While miR-22-3p is known to be involved in cardiac hypertrophy and remodeling, SIRT1 is an important director of the metabolic response to fasting in mammals; while its overexpression reduces insulin resistance and promotes lipid mobilization in adipocytes [79].

Unfortunately, no adequate data exist to fully understand all of the molecular processes involved in the observed effects of PDE5i in T2DM. For example, the continued excess of glucose release by the liver and beta-cell dysfunction play important roles in the pathogenesis of T2DM [80, 81]. Accordingly, a study conducted in rat liver cells demonstrated that sildenafil decreased liver glycogenolysis and gluconeogenesis; in particular, treatment with a high dose of PDE5i (5 mg/kg) reduced the hepatic glycogen phosphorylase (GP), which breaks up glycogen into glucose subunits, without affecting the phosphoenolpyruvate carboxykinase enzyme, which participates in gluconeogenesis in animal tissues [82].

Moreover, studies performed in patients affected by metabolic syndrome reported that the administration of tadalafil could improve beta-cell function. Interestingly, the authors showed a significant effect after tadalafil treatment only in female patients who demonstrated more sensitivity to cGMP stabilization than males [83, 84]. In a double-blind placebo-controlled study of T2DM patients randomized to receive sildenafil (100 mg/d for 12 weeks), a reduction of waist circumference (WC) and a reduction of the epicardial adipose tissue (EAT) were observed and compared to placebo, without changes in body weight or body mass index (BMI) [79].

The results of these studies underline the potential usefulness of PDE5 inhibition in T2DM, not only for metabolic control but also for the associated inflammatory status and vascular damage.

## 6. Conclusions

Besides their well-known effects on ED, different preclinical and clinical studies on the use of PDE5 inhibitors for metabolic disease treatment have been conducted. Indeed, PDE5 enzymes are highly expressed in all insulin-sensitive tissues, such as endothelium, muscle, adipocytes, and hepatocytes. Moreover, PDE5i interfere with the NO/cGMP signaling, an important pathway which controls glucose metabolism regulation. Thus, by regulating NO/cGMP pathways, PDE5i administration could provide a good strategy to promote glucose uptake and may be a new pharmacological approach to treat metabolic diseases (Figure 2).

Given the safety record of these drugs, it is desirable to speculate on the use of PDE5 inhibition as a potential approach in the prevention of diet-induced IR and metabolic imbalances. Although different encouraging data are available, further in vivo and in vitro indications are mandatory.
